# Determining the number of hidden layer and hidden neuron of neural network for wind speed prediction

**DOI:** 10.7717/peerj-cs.724

**Published:** 2021-09-20

**Authors:** Muhammad Ibnu Choldun Rachmatullah, Judhi Santoso, Kridanto Surendro

**Affiliations:** School of Electrical Engineering and Informatics, Institut Teknologi Bandung, Bandung, West Java, Indonesia

**Keywords:** Neural network, Hidden layer, Hidden neuron, PCA, K-means clustering, Regression, Wind speed prediction

## Abstract

Artificial neural network (ANN) is one of the techniques in artificial intelligence, which has been widely applied in many fields for prediction purposes, including wind speed prediction. The aims of this research is to determine the topology of neural network that are used to predict wind speed. Topology determination means finding the hidden layers number and the hidden neurons number for corresponding hidden layer in the neural network. The difference between this research and previous research is that the objective function of this research is regression, while the objective function of previous research is classification. Determination of the topology of the neural network using principal component analysis (PCA) and K-means clustering. PCA is used to determine the hidden layers number, while clustering is used to determine the hidden neurons number for corresponding hidden layer. The selected topology is then used to predict wind speed. Then the performance of topology determination using PCA and clustering is then compared with several other methods. The results of the experiment show that the performance of the neural network topology determined using PCA and clustering has better performance than the other methods being compared. Performance is determined based on the RMSE value, the smaller the RMSE value, the better the neural network performance. In future research, it is necessary to apply a correlation or relationship between input attribute and output attribute and then analyzed, prior to conducting PCA and clustering analysis.

## Introduction

The energy requirement continuously grows as the world population increases. Such energy requirement increases sometimes is not accompanied with the increase of supporting facilities and infrastructure development making several locations do not obtain sufficient electricity input. This case encourages the utilization of renewable energy in order to meet the world energy demand in various sectors including agriculture, education, health, road lighting, and community economy driving force (Jamil & Zeeshan, 2019; Zhang et al., 2019). Wind-based energy has long been utilized in irrigation sector, while other sources reveal that wind energy was firstly used in India (Mathew, 2006). Although it has long been used in various sectors, wind speed prediction does not belong to an easy work due to its high strong randomness and volatility, whereas accurate wind speed prediction is needed in our life. One of the important factors in predicting wind speed is its accuracy (Peiris, Jayasinghe & Rathnayake, 2021; Yadav, Muneender & Santhosh, 2021). As an example, the accuracy of wind speed prediction is essential in terms of wind power plant (Zhang et al., 2019).

Several different techniques have been used to predict the wind speed, including physical method (Lange & Focken, 2009; Li et al., 2013), statistical method, and combination method between them. The use of physical method can be seen in the use of Computational Fluid Dynamics (CFD), where such approach does not depend on the historical data and can be used wider in all kinds of wind power plant including the newest wind power plant (Li et al., 2013). The use of statistical method is in the form of the use of auto regressive model (AR), moving average model (MA), autoregressive moving average model (ARMA), and auto regressive integrate moving average model (ARIMA) (Lei et al., 2009). In addition to these two methods, neural network is recently often used to predict the wind speed (Jamil & Zeeshan, 2019; Madhiarasan & Deepa, 2016; Madhiarasan & Deepa, 2017; Zhang et al., 2019). The combination of the existing methods is also often used to predict the wind speed, such as the use of autoregressive fractionally integrated moving average and improved back-propagation neural network (Wang & Li, 2019).

Artificial neural networks, as a part of artificial intelligence methods have been widely used in many fields for prediction purposes(Bakhashwain & Sagheer, 2021; Rahman et al., 2021; Zhao & Liu, 2021), including wind speed prediction. One of the crucial factor for designing a neural network is its structure or topology, namely determining the hidden layers number and the hidden neurons number for corresponding hidden layer because it is closely related to the topological performance (Aggarwal, 2018; Koutsoukas et al., 2017; Nitta, 2017), but until now topology determination is still a complex and difficult problem (Lee et al., 2018; Naitzat, Zhitnikov & Lim, 2020; Rahman et al., 2021). Topology is one of the important hyperparameters in neural networks. Determining the topology that does not match the needs caused overfitting or underfitting in neural networks. Several researchers have conducted research to determine the neural network topology in various ways: methods based solely on the number of input and output attributes (Sartori & Antsaklis, 1991; Tamura & Tateishi, 1997), trial and error (Blanchard & Samanta, 2020; Madhiarasan, 2020; Madhiarasan & Deepa, 2016; Madhiarasan & Deepa, 2017; Şen & Özcan, 2021) , and the rule of thumb (Bakhashwain & Sagheer, 2021; Carballal et al., 2021; Rahman et al., 2021).

In this research, the determination of the neural network topology use PCA and K-means clustering (Rachmatullah, Santoso & Surendro, 2020), but for the new objective function. Whereas in the previous research (Ibnu Choldun, Santoso & Surendro, 2020; Rachmatullah, Santoso & Surendro, 2020) the determination of the neural network topology was used for the classification objective function, in this research it was used for the regression objective function, specifically to predict wind speed. The scientific major contribution of this research is the use of a new method to determine the neural network topology using PCA and clustering for the regression objective function. The main difference is that the attribute classification objective function is categorical while the output regression objective function must be numeric. The performance measurement is also different, if the classification uses the accuracy rate, while if the regression uses error rate. The purpose of this research is to perform regression, so that the cumulative variance required is expected to be greater than classification, because the output domain for regression is continuous, while for classification is discrete. Then, topology performance of neural network in this research was compared with several other methods, namely: the Sartori method (Sartori & Antsaklis, 1991), the Tamura and Tateishi method (Tamura & Tateishi, 1997), the Madhiarasan and Deepa method(Madhiarasan & Deepa, 2017), the Madhiarasan method (Madhiarasan, 2020), and the Mahdi method (Mahdi, Yousif & Melhum, 2021).

The next section of this paper is structured as follows. The ‘Materials & Methods’ section contains the methodology of the proposed method starting from the data preparation to the topology evaluation. The ‘Results and Discussion’ section explains the results of the experiment and its discussion, especially about topology determination and topology evaluation. The ‘Conclusions and Future Work’ concludes and proposes future works containing a summary of the results of this study and provide direction for subsequent research studies.

## Materials & Methods

**Figure 1:**
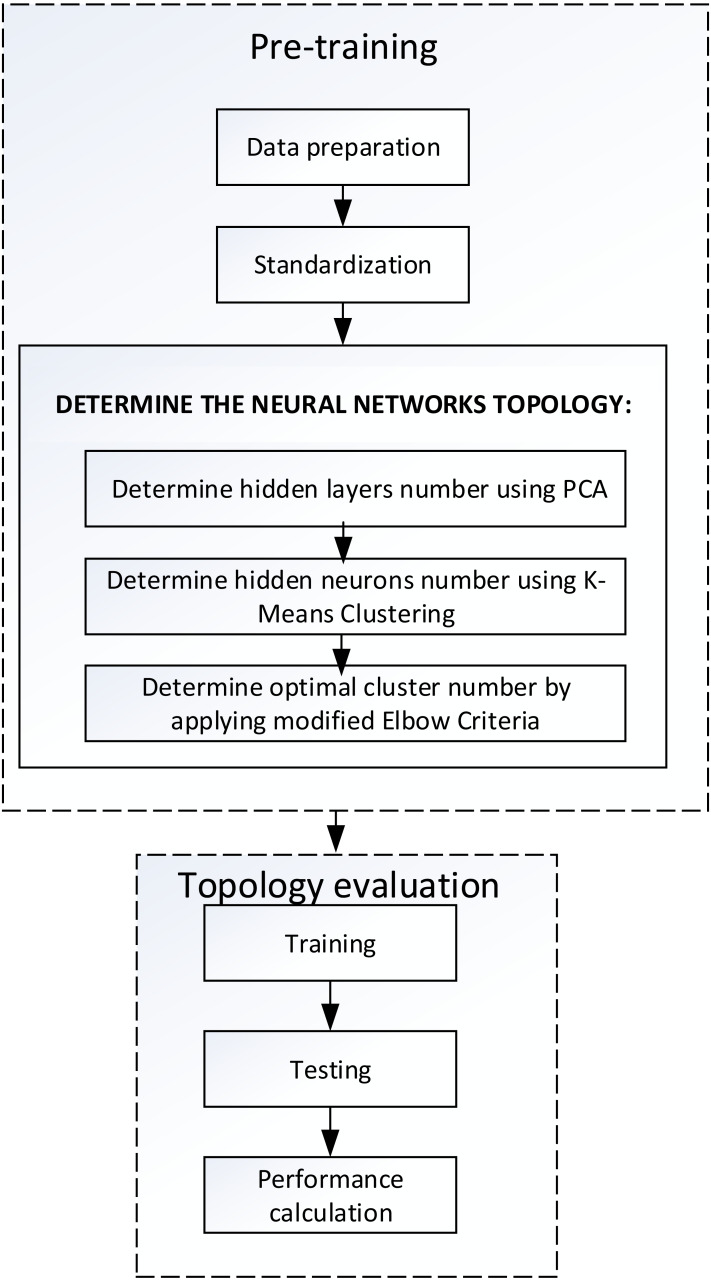
The methodology of proposed method.

The methods is presented with a clear outline as illustrated in Fig. 1. In general, the methods is divided into two main steps, namely the pre-training and the topology evaluation. The pre-training step was conducted before the model formation process of learning including preparation or selection of datasets, pre-processing data, and measurement of the topology of neural networks. Stages of topology evaluation were applied to manage the learning performance of neural networks based on the topology. The stage involves training, testing, and performance calculation. The proposed method focuses on determining the topology of neural networks for regression objective function which includes three main steps, namely:

1. Analyzing the dataset by applying PCA, therefore it can obtained significant principal components.
2. Performing clustering using the K-means technique for each corresponding principal component by changing the clusters number
3. Determining the optimal clusters number for each corresponding principal component by applying Elbow criteria, so that the optimal clusters number is obtained for each corresponding principal component.

Each stage will be explained in the next section.

### Data preparation

This study aims to predict wind speed, so researchers choose a dataset providing attributes to predict wind speed. The dataset was meteorological data (London Meteorological data) downloaded from http://www.urban-climate.net/content/data/9-data for 2016 consisting of 8784 data. This dataset had many features, but researchers only selected attributes related to wind speed prediction. These attributes are input and output as in Table 1:

**Table 1:** Attribute used.

No.	Attribute name	Input/ Output	Range	Mean
1	CR10 temperature	Input	11.140–29.720	18.591
2	Last minute average temperature	Input	−1.267–32.400	12.413
3	Maximum hourly air temperature	Input	−1.172–33.480	12.809
4	Minimum hourly air temperature	Input	−1.280–31.540	12.063
5	Wind speed 10 minutes	Output	0.000–28.250	7.786

Table 1 explains there are four input attributes, including CR10 Temperature, Last Minute Average Temperature, Maximum Hourly Air Temperature, and Minimum Hourly Air Temperature. These attributes have a role to predict the output attribute (Wind Speed). The range of values for these five attributes can be seen in column 4, while, the average can be seen in column 5.

### Standardization

Standardization utilizes the normalization process so that data can be obtained with the consistent scale attributes. The normalization used is the Min-Max with a value between 0 and 1 using Eq. (1) (Dharamvir, 2020). The formula for normalization with Min-Max technique is as follows:

(1) \begin{eqnarray*}{d}^{{}^{{^{\prime}}}}= \frac{d-{\min \nolimits }_{1}}{ma{x}_{1-}{\min \nolimits }_{1}} \left( {\max \nolimits }_{2}-{\min \nolimits }_{2} \right) +{\min \nolimits }_{2}\end{eqnarray*}

d’ = the new value of data, d = old data, min_1_ = the lowest value of corresonding attribute, max_1_ = the highest of corresponding attribute, min_2_ = 0, new_max_2_ = 1.

After reaching the normalization stage with min-max (0–1), Table 2 shows that the five attributes now have the same range between 0 and 1 as shown in the fourth column and the average value shown in the fifth column.

**Table 2:** Normalized attributes.

**No.**	**Attribute name**	**Input/ Output**	**Range**	**Mean**
1	CR10 temperature	Input	0.000–1.000	0.401
2	Last minute average temperature	Input	0.000–1.000	0.406
3	Maximum hourly air temperature	Input	0.000–1.000	0.403
4	Minimum hourly air temperature	Input	0.000–1.000	0.407
5	Wind speed 10 minutes	Output	0.000–1.000	0.276

### Determining of neural network topology

**Figure 2:**
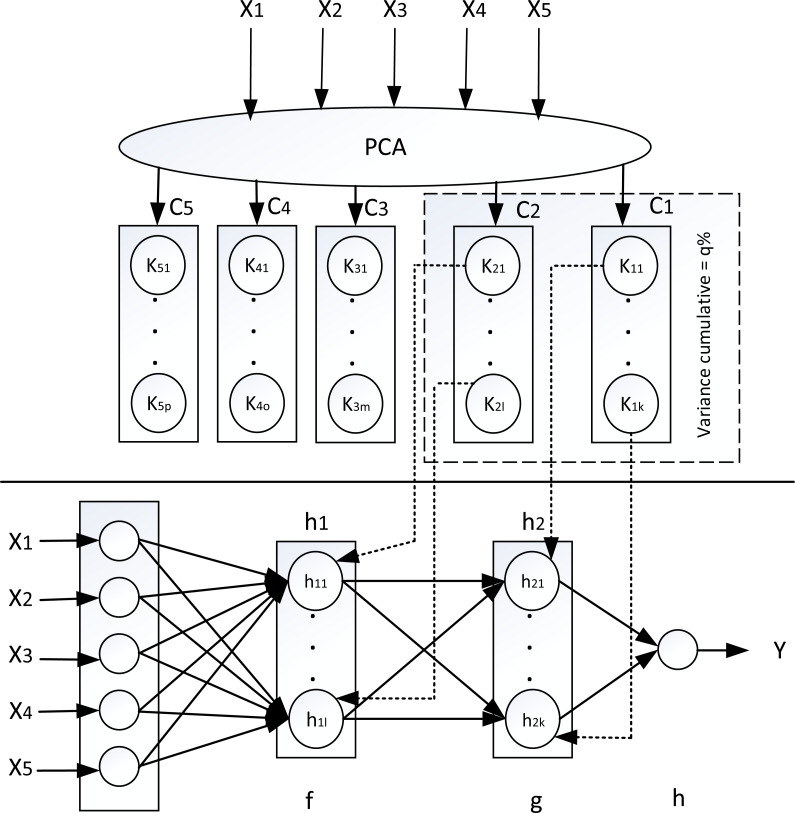
Determine neural netwoks topology for regression using PCA and clustering.

In this research, the determination of the neural network topology to predict wind speed is based on previous research (Ibnu Choldun, Santoso & Surendro, 2020; Rachmatullah, Santoso & Surendro, 2020) that uses PCA and clustering with the K-means technique as illustrated in Fig. 2, but for the regression objective function.

In a neural network, increasingly complex features represent increasingly higher information content. Meanwhile, the high content of information in PCA is represented in the principal component which has a high variance. Therefore, the hidden layers that have more complex features are consistent with the PCA components that have higher variance. Accordance with this rationale, the hidden layer number in neural networks needed is consistent with the principal components number in PCA. Hence in this research, the determination of hidden layer number in neural networks based on principal components number obtained through PCA. This determination is accordance with the consideration that the PCA cumulative variance is compatible with the complexity of hidden layer in neural network as in Eq. (2).

(2) \begin{eqnarray*}\sum Variance \left( P{C}_{i} \right) \approx \sum Complexity({h}_{i})\end{eqnarray*}

PC_i_ is PCA component and h_i_ is the neural network hidden layer

So for example in the figure above, a dataset that has four attributes of input, after a principal component analysis is carried out, there will be four principal components where the principal component equation is a input attributes linear combination (Liu & Ding, 2020; Ratner, 2017). Since the input attributes number is four, the principal component equation is as follows:

(3) \begin{eqnarray*}P{C}_{1}& ={w}_{11}{x}_{1}+{w}_{12}{x}_{2}+{w}_{13}{x}_{3}+{w}_{14}{x}_{4}+{w}_{15}{x}_{5}\nonumber\\\displaystyle P{C}_{2}& ={w}_{21}{x}_{1}+{w}_{22}{x}_{2}+{w}_{23}{x}_{3}+{w}_{24}{x}_{4}+{w}_{25}{x}_{5}\nonumber\\\displaystyle P{C}_{3}& ={w}_{31}{x}_{1}+{w}_{32}{x}_{2}+{w}_{33}{x}_{3}+{w}_{34}{x}_{4}+{w}_{35}{x}_{5}\nonumber\\\displaystyle P{C}_{4}& ={w}_{41}{x}_{1}+{w}_{42}{x}_{2}+{w}_{43}{x}_{3}+{w}_{44}{x}_{4}+{w}_{45}{x}_{5}\end{eqnarray*}

w = weight, x_i_ = ith input attribute

Of the four components, for example, only two principal components that have a cumulative variance q% were selected (Yang, 2019). The two principal components selected with the cumulative variance q% are the basis for determining the hidden layers number in neural network, namely using two hidden layers. Then we clustered each selected component using K-means clustering(Alguliyev, Aliguliyev & Sukhostat, 2020; Hancer, Xue & Zhang, 2020). The optimal clusters number for corresponding principal component was determined using the Elbow criteria (Shmueli et al., 2020). The optimal clusters number for each component is the basis for determining the hidden neurons number in corresponding hidden layer on the neural network.

### Topology evaluation

The training process was carried out with the amount of data as much as 70% of the dataset, while testing was carried out with 30% of the data from the dataset (Nguyen et al., 2021). Both the training and testing processes for each topology were repeated ten times by varying the initial weight values. For the regression objective function, the following performance measures can be used: Mean Absolute Percentage Error (MAPE), Mean Absolute Error (MAE), Root Mean Square Error (RMSE), or Mean Square Error (MSE). MAPE and MAE are suitable for time series datasets. In this research, the dataset used is not time series data so that the performance measurement was selected using RMSE (Namasudra, Dhamodharavadhani & Rathipriya, 2021). Calculation of the topology performance of neural network is based on the value of the root mean of squared error (RMSE) with the formula:

(4) \begin{eqnarray*}RMSE=\sqrt{ \frac{1}{N} \sum _{i=1}^{n}(\widehat{{y}_{i}}-{y}_{i})^{2}}\end{eqnarray*}

where, N: the number of data, $\widehat{{y}_{i}}$: the prediction value, y_i_: the target value. Lower RMSE presents higher performance.

To compare the topology performance based on PCA and clustering, it is necessary to compare with several other methods. Some of these methods are:

1. Sartori method (Sartori & Antsaklis, 1991) used one hidden layer with

(5) \begin{eqnarray*}\mathrm{The~ neuron~ number}=\mathrm{N}-1\nonumber\\\displaystyle \mathrm{N}=\mathrm{the~ input~ feature~ number}.\end{eqnarray*}

2. Tamura and Tateishi method (Tamura & Tateishi, 1997) used two hidden layers, where

(6) \begin{eqnarray*}\mathrm{The~ neuron~ number~ of~ the~ corresponding~ hidden~ layer : }~\mathrm{N}/2+3\nonumber\\\displaystyle \mathrm{N}=\mathrm{the~ number~ of~ input~ feature}.\end{eqnarray*}

3. Madhiarasan and Deepa method (Madhiarasan & Deepa, 2017) used one hidden layer and through trial and error, it was found that the number of neurons was 14.

4. Madhiarasan method (Madhiarasan, 2020), used one hidden layer and through trial and error, it was found that the number of neurons was 44.

5. Mahdi et al. method (Mahdi, Yousif & Melhum, 2021) used one hidden layer and through trial and error, it was found that the number of neurons was 20.

To calculate the performance of the topology we use the Windows 10 operating system and Rapidminer 9.5 tools.

## Results and Discussion

In this section, the process of determining the neural network topology will be presented as a result of the application of principal component analysis(PCA), clustering with K-means method and the application of modified Elbow criteria to the wind dataset.

### Determine the topology of neural networks using PCA and K-means clustering

After normalization, both input attributes and output attributes have a value range between 0 and 1 as presented in Standardization section. Principal component analysis was done on the four normalized input attributes.. The PCA results can be seen in the second and third columns of Table 3. The first PCA principal component has a highest variance which is of 0.943 (94.3%) so that the cumulative variance is also 0.943. The second principal component of PCA has a a second highest variance which is 0.056 (5.6%) so the cumulative variance is 0.943 + 0.056 = 0.99 9. The variance for each main component was obtained from the proportion between the eigenvalues of a component and the total eigenvalues of all components. Likewise for the third component and fourth component can be seen in the second and third columns of Table 3.

**Table 3:** PCA and clustering of the wind dataset.

**Component**	**Variance**	**Cumulative**	**Clustering**
PC 1	0.943	0.943	10
PC 2	0.056	0.999	3
PC 3	0.001	1.000	2
PC 4	0.000	1.000	2

For each component that has been generated from the PCA process, clustering is carried out so that the optimal clusters number of clusters is obtained. The clustering application is carried out using the K-means method, while to determine the optimal cluster number using the modified Elbow criteria. The optimal clusters number has been obtained based on the wss value, that is, when the wss value in a row (at least three in a row) has remained relatively unchanged (Rachmatullah, Santoso & Surendro, 2020). The examples of applying the modified Elbow criteria are shown in Table 4, which is the result of applying these criteria to the first PCA principal component that has the highest variance. Hence, the number of clusters gradually increased from 2 to 50, while the wss value calculation results appeared. At *N* = 10, 11, and 12 the value of the three wss values in a row does not change, so it can be concluded that the number of neurons for the first component is 10.

**Table 4:** The result of application of K-means clustering and modified elbow criteria for the first component.

N	wss	N	wss	N	wss	N	wss
2	0.035	15	0.001	27	0.000	39	0.000
3	0.019	16	0.001	28	0.000	40	0.000
4	0.012	17	0.001	29	0.000	41	0.000
5	0.008	18	0.001	30	0.000	42	0.000
6	0.006	19	0.001	31	0.000	43	0.000
7	0.004	20	0.001	32	0.000	44	0.000
8	0.003	21	0.001	33	0.000	45	0.000
9	0.003	22	0.000	34	0.000	46	0.000
10	0.002	23	0.000	35	0.000	47	0.000
11	0.002	24	0.000	36	0.000	48	0.000
12	0.002	25	0.000	37	0.000	49	0.000
13	0.001	26	0.000	38	0.000	50	0.000
14	0.001						

The results of K-means clustering and applying the modified Elbow criteria for corresponding component can be seen in the fourth column of Table 3. For example, for the PCA first component the optimal clustersnumber is 10, for the PCA second component the optimal clusters number is 3, and so on with the same explanation.

**Figure 3:**
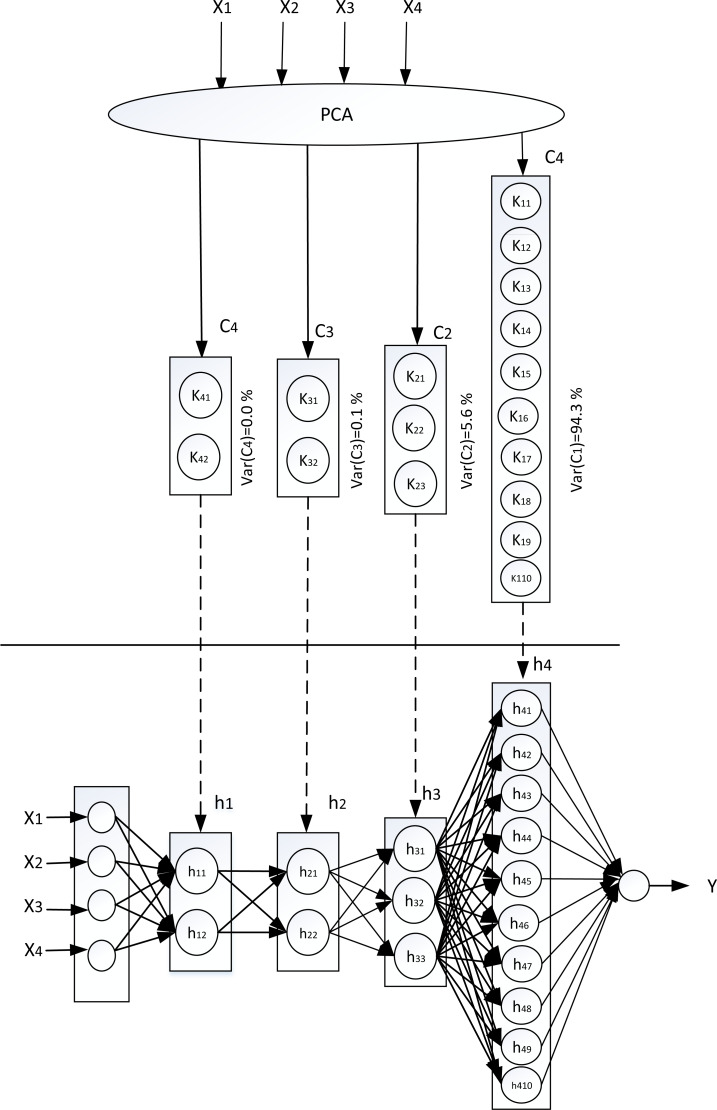
Mapping PCA to neural network. (X_1_ = CR10 temperature, X_2_ = Last minute average temperature, X_3_ = Maximum hourly air temperature, X_4_ = Minimum Hourly air temperature, Y = Wind speed 10 minutes).

The results obtained from the PCA process and K-means clustering were used in determining the neural network topology. The determination of this topology consists of determining the hidden layers number and the neurons number in corresponding of these hidden layers. Since it had reached the cumulative variance of 90% using one component, the topology of the neural network was evaluated using one hidden layer to four hidden layers. As an explanation, if one component is to be used which has the optimal number of clusters is 10, then the topology is to use one hidden layer with the neurons number is 10; if two components are to be used, then the hidden layers number is two in which the first hidden layer has 3 neurons and meanwhile the second hidden layer has 10 neurons; and so on in the same explanation as shown in Fig. 3. This figure shows the topology that use four hidden layers. The first principal component of PCA, which has the highest variance, corresponds to the hidden layer closest to the output layer.

### Performance comparison

The performance comparison of the topology of neural network from the results of PCA and clustering, it needs analysis of each of the topologies and a comparison with five methods proposed by other researchers. The Sartori method and the Tamura and Tateishi method with *N* = 4 (input attribute number) respectively get topology (3) and topology (5,5). Three other methods are the Madhiarasan and Deepa methods, the Madhiarasan method, and the Mahdi method which uses one hidden layer sequentially using topology (14), topology (44), and topology (20). These five method proposed by the other researchers is used as a comparison with the the method used by researchers.

Table 5 presents the RMSE mean of each topology determined by PCA process and K-means clustering, where each topology is applied to the process of learning using a neural network with 100 cycles. For each topology, each experiment was repeated ten times by varying the seed. The column “Topology” presents the hidden layers number and the neurons number for each topology. As an explanation, column “3,10” shows a topology consisting of two hidden layers with the first hidden layer has 3 neurons and the second hidden layer has 10 neurons. The values in the table shows the RMSE value, while the bottom row present the mean of RMSE from 10 repetitions.

**Table 5:** The RMSE value of topology with cycles is 100.

**No.**	**Seed**	**Topology**			
		10	3,10	2,3,10	2,2,3,10
1	0	0.1284	0.1266	0.1299	0.1534
2	1	0.1197	0.12	0.1199	0.1424
3	2	0.1522	0.1519	0.1524	0.1938
4	3	0.1484	0.1546	0.1562	0.1626
5	4	0.1206	0.1212	0.1220	0.1430
6	5	0.1607	0.1533	0.1541	0.1748
7	10	0.1288	0.1266	0.1274	0.1494
8	15	0.1198	0.1196	0.1196	0.1387
9	20	0.1311	0.1292	0.1301	0.1433
10	25	0.1294	0.131	0.1255	0.1384
	**Mean**	**0.13391**	**0.1334**	**0.13371**	**0.15398**

**Figure 4:**
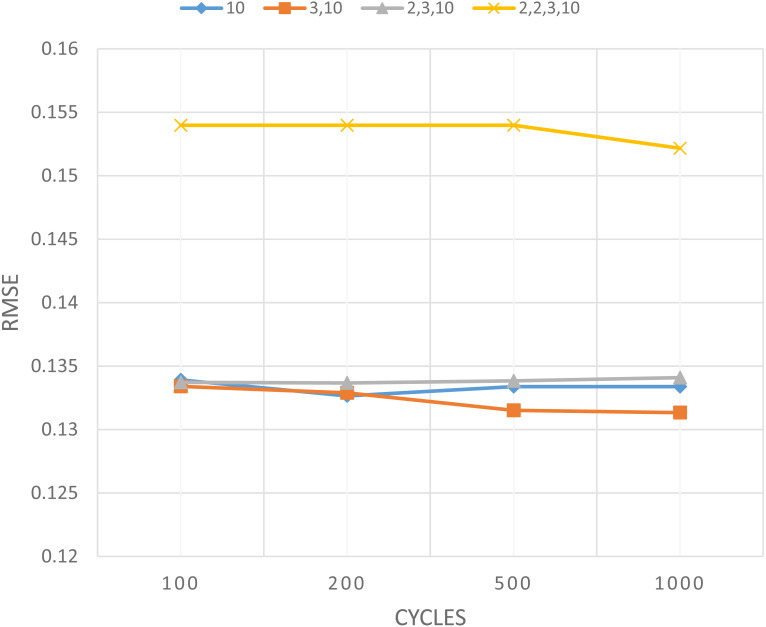
RMSE value for topology determined using PCA and clustering.

In the same way, the experiments in Table 5 also implement some different cycles, including 200 cycle, 500 cycle, and 1,000 cycle. The summary that shows the mean value of each cycles is displayed in Table 6, while in graphical form is manifested in Fig. 4. The values in Table 6 explains the average RMSE value of 10 repetitions for each topology, the details of which can be worked out following Table 5. Through the same experiment, Table 6 were also carried out for the number of cycles of 200 cycle, 500 cycle, and 1,000 cycle.

**Table 6:** RMSE mean of topology (PCA and K-means clustering).

**Cycles**	**Topology**			
	10	3,10	2,3,10	2,2,3,10
100	0.13391	0.1334	0.13371	0.15398
200	0.13265	0.1329	0.13367	0.15398
500	0.13339	0.13151	0.13384	0.15398
1000	0.13338	0.13133	0.13409	0.15217

**Figure 5:**
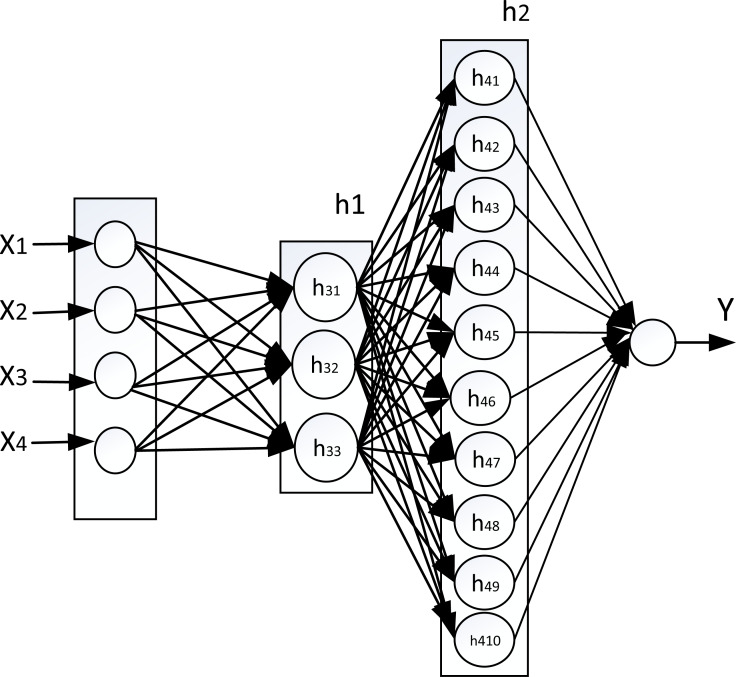
The original topology which has the best performance. (X_1_ = CR10 temperature, X_2_ = Last minute average temperature, X_3_ = Maximum hourly air temperature, X_4_ = Minimum hourly air temperature, Y = Wind speed 10 minutes).

In Fig. 4, the horizontal axis is the cycles number, and the vertical axis is the mean value of RMSE. The graph presents the mean of RMSE values for each topology in each cycle. The graph in Fig. 4 shows the topology (3,10) has a tendency of RMSE mean values lower than other topologies, then followed by topology (10), topology (2,3,10) and topology (2,2,3,10). The graph also presents the addition of the hidden layers number does not provide a guarantee to reduce the RMSE value. Based on the topology from PCA and clustering, it has two hidden layers, which gives the lowest RMSE value, so it proves the best performance of the topology. Mapping from PCA and clustering into the topology of neural network gives the best performance, namely topology (3,10) as shown in Fig. 5. This topology that uses two hidden layers requires a cumulative variance of 99%, so that the selected topology will be compared with the topology of other researchers. This also proves that the cumulative variance PCA required for the regression objective function is greater than the classification objective function as has been done in previous research: for multi-class classification it needs a PCA cumulative variance of about 70% (Ibnu Choldun, Santoso & Surendro, 2020; Rachmatullah, Santoso & Surendro, 2020) while for binary classification it needs a PCA cumulative variance of about 40% (Rachmatullah, Santoso & Surendro, 2020).

In this research we also processes the dataset as listed in Table 1 using the topologies that are compared so that each topology has an RMSE value. Table 7 shows the experiment results on the dataset using the method used by researchers and five other methods used by other researchers. This table presents the comparison of the RMSE between all topologies. Each topology is applied to the learning process using a neural network with 100 cycles.

**Table 7:** The comparison of topology RMSE value with cycles is 100.

**No.**	**Seed**	**Topology**					
		Researcher (3,10)	Sartori (3)	Tateishi& Tamura (5,5)	Madhiarasan& Deepa (14)	Madhiarasan (44)	Mahdi (20)
1	0	0.1266	0.1378	0.1264	0.1287	0.1283	0.1287
2	1	0.1200	0.1262	0.1202	0.1198	0.1194	0.1198
3	2	0.1519	0.1500	0.1525	0.1512	0.1508	0.1515
4	3	0.1546	0.1475	0.1545	0.1485	0.1531	0.1485
5	4	0.1212	0.1312	0.1218	0.1208	0.1196	0.1196
6	5	0.1533	0.1533	0.1520	0.1618	0.1597	0.1608
7	10	0.1266	0.1418	0.1266	0.1287	0.1285	0.1291
8	15	0.1196	0.1309	0.1196	0.1197	0.1196	0.1200
9	20	0.1292	0.1415	0.1289	0.1306	0.1294	0.1309
10	25	0.1310	0.1286	0.1314	0.1282	0.1193	0.1296
	**Mean**	**0.1334**	**0.13888**	**0.13339**	**0.1338**	**0.13277**	**0.13385**

**Figure 6:**
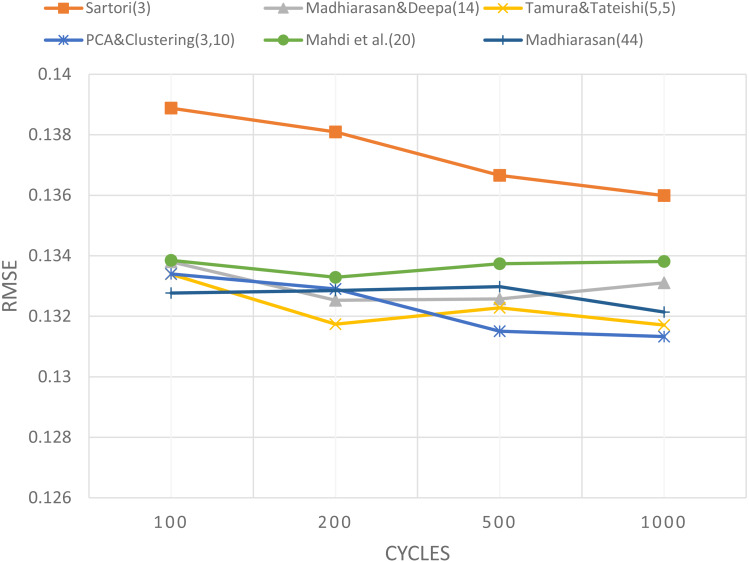
The comparison of the RMSE value between topology used by researchers and other topologies.

The presentation of Table 7 is the same as the presentation of Table 5 as previously explained and also implementedin some different cycles, including 200 cycle, 500 cycle, and 1,000. The summary that shows the mean the mean values of each cycle is as shown in Table 8, whereas the graph can be seen in Fig. 6. The horizontal axis, vertical axis, and graphs for Fig. 6 are explained in the same way as Fig. 4. Figure 6 exposes the RMSE value for comparison between the topology used by researcher(PCA and Clustering method) and the five topologies that used by other researchers, namely: the Sartori method, the Tamura and Tateishi method, the Madhiarasan and Deepa method, the Madhiarasan method, and Mahdi method.

**Table 8:** RMSE mean of topology (PCA + clustering) and other topology.

**Cycles**	**Topology**					
	**Researcher** **(3,10)**	**Sartori** (3)	**Tamura& Tateishi (5,5)**	**Madhiarasan& Deepa (14)**	**Madhiarasan** (44)	**Mahdi** (20)
100	0.13340	0.13888	0.13339	0.13380	0.13277	0.13385
200	0.13290	0.13809	0.13174	0.13253	0.13285	0.13329
500	0.13151	0.13666	0.13228	0.13257	0.13298	0.13374
1000	0.13133	0.13599	0.13171	0.13311	0.13214	0.13381

In Fig. 6, the horizontal axis is the cycles number, and the vertical axis is the mean value of RMSE. The graph presents the mean of RMSE values for each topology in each cycle. The graph in Fig. 6 shows the topology used by researchers has a tendency of RMSE mean values lower than other topologies, then followed by Tamura and Tateishi topology (5,5), Madhiarasan (44), Madhiarasan and Deepa topology (14), Mahdi topology (20), and Sartori topology (3). The graph also shows that the using two hidden layers tends to have a lower RMSE than using only one hidden layer. The topology used by researchers based on PCA and clustering with two hidden layers, which gives the lowest RMSE value, so this proves that this topology has the best performance compared to the topologies used by other researchers.

Patterson and Gibson proposed to provide hidden neurons in large numbers in the network so that the performance of neural networks is better. But network performance can be degraded when the number of neurons is too large because it may have several false connections (Patterson & Gibson, 2017). For example, using a higher number of neurons in Madhiarasan (44) than in Madhiarasan and Deepa topology (14), Mahdi topology (20), and Sartori topology (3), can improve the neural networks performance. However, increasing the number of neurons using only one hidden layer does not always guarantee an increase in performance, such as the performance of Madhiarasan (44) which is lower than the topology that uses two hidden layers with fewer neurons, the Tamura & Tateishi (5.5) topology and the proposed topology (3.10). This study also shows that the the cumulative variance for the regression objective function, in this study 99% greater than the cumulative variance for the classification objective function in previous studies (Rachmatullah, Santoso & Surendro, 2020), where for binary classification needs a PCA cumulative variance of 38.9%, while the multi-class classification needs a PCA cumulative variance of 69.7%.

## Conclusions and Future Work

In this research paper, performance analysis of various neural network is compared to predict the wind speed. Comparison was made between the PCA and clustering method and several other methods. The PCA and clustering method uses PCA to set the hidden layers number, whereas K-means clustering of these components formed from PCA is used to determine the optimal clusters number used as a guidance to set the neurons number in corresponding hidden layer. The experimental results report that the topology originating from PCA and clustering has a fairly good performance compared to other methods by looking at the mean value of RMSE. The topology of Neural network determination using PCA and clustering can provide optimal performance.

In future research, it is necessary to apply a correlation or relationship between input attributes and output attributes and then analyzed, prior to conducting PCA and clustering analysis. Variations in input attributes also need to be analyzed before implementing PCA and K-means clustering. Considering correlation of attributes and the variation of attributes is expected to produce a topology of neural network design that has better performance. Future researches can also use other clustering methods to determine the number of neuron.

## Supplemental Information

10.7717/peerj-cs.724/supp-1 Supplemental Information 1 London Meteorological data, 2016

10.7717/peerj-cs.724/supp-2 Supplemental Information 2 The selected attributes for wind speed prediction This dataset had many features, but researchers only selected attributes related to wind speed prediction. There are four input attributes, including CR10 Temperature, Last Minute Average Temperature, Maximum Hourly Air Temperature, and Minimum Hourly Air Temperature. These attributes have a role to predict the output attribute (Wind Speed).

10.7717/peerj-cs.724/supp-3 Supplemental Information 3 Source code for wind speed prediction using Rapidminer This source code can be changed according to the experiments to be carried out: Line 63 is changing the local random seed as desired The number of hidden layers and the number of neurons for each hidden layer can be changed on lines 67 and 68 line 67 line 68 The number of cycles used can be changed on the 70 line 70.

## Additional Information and Declarations

### Data Availability

The following information was supplied regarding data availability: The London Meteorological data for 2016 is available from: http://www.urban-climate.net/content/data/9-data.
